# Recurrent Wheezing and Asthma After Respiratory Syncytial Virus Bronchiolitis

**DOI:** 10.3389/fped.2021.649003

**Published:** 2021-06-04

**Authors:** Yunlian Zhou, Lin Tong, Mengyao Li, Yingshuo Wang, Lanxin Li, Dehua Yang, Yuanyuan Zhang, Zhimin Chen

**Affiliations:** Department of Pulmonology, Children's Hospital, Zhejiang University School of Medicine, National Clinical Research Center for Child Health, Hangzhou, China

**Keywords:** asthma, bronchiolitis, respiratory syncytial virus, recurrent wheezing, children

## Abstract

**Background:** Respiratory syncytial virus (RSV) is the most common pathogen of acute bronchiolitis in children, which sometimes triggers the development of recurrent wheezing and increases the risk of childhood asthma.

**Methods:** We enrolled 425 children who were diagnosed with RSV-infected bronchiolitis at the department of pulmonology, Children's Hospital Zhejiang University School of Medicine in 2011. Long-term follow-up was performed to explore the consequence of bronchiolitis on subsequent recurrent wheezing and asthma.

**Results:** Of 425 patients, 266 cases completed the entire follow-up, the mean age of onset was 4.9 (3.3) months, and the male-to-female ratio was 2.5. The mean birth weight of all patients was 3.22 (0.63) kg, and the number of patients who had a history of cesarean section was 148. According to the outcome of follow-up, 36 were in the recurrent wheezing (RW) group, 65 were in the asthma (AS) group, and the remaining 165 were in the completely recovered (CR) group. The age of onset was older and the birth weights were higher in the AS group than those in the CR group (*P* < 0.05). And the higher proportion of cesarean sections was higher in the RW group than that in the CR group (*P* < 0.05). Furthermore, we found a remarkable increasing of serum IgE in the AS groups than that in the CR group (*P* < 0.01). Multiple logistic regression analysis showed that the cesarean section was the risk factor for the development of recurrent wheezing and the higher birth weight was the risk factor for the development of asthma.

**Conclusion:** RSV bronchiolitis might increase the incidence of recurrent wheezing and asthma. Allergic constitution was an important prerequisite for the occurrence of asthma, and related risk factor such as cesarean section can only increase recurrent wheezing to a certain extent within a certain period of time. And we also find higher birth weight and older onset age for those who develop asthma, which should be verified in the future.

## Introduction

Acute bronchiolitis caused by respiratory syncytial virus (RSV) is a common infectious disease, which is generally believed that almost 100% of children within the first 2 years of life have infected with RSV (1). The typical clinical manifestations include cough, wheezing, shortness of breath, dyspnea, etc. In severe cases, it can lead to respiratory failure and even life-threatening. Moreover, RSV infection could damage the airway and led to frequent and persistent wheezing in children (2, 3), and some researchers even believed that it might increase the incidence of asthma (4, 5).

The relationships between RSV bronchiolitis and recurrent wheezing/asthma have been reported by many studies (6). However, it is well-known that asthma is a heterogeneous disease, which is affected by genes, environment and the level of economics, which would lead to regional differences in asthmatic incidence. And there were few long-term follow-up studies to analyze the impact of RSV infection on recurrent wheezing and asthma in China. So, in this study we enrolled 425 patients who were diagnosed with RSV-infected bronchiolitis in our hospital during 2011, collected clinical data and conducted follow-up for up to 7 years to explore the effects of RSV infection on the subsequent recurrent wheezing and even asthma.

## Materials and Methods

### Participants

We prospectively and consecutively enrolled patients under 2 years old who were admitted to the department of pulmonology at Children's Hospital, Zhejiang University School of Medicine in 2011 for bronchiolitis caused by RSV. Bronchiolitis was diagnosed according to the clinical practice guideline on the diagnosis, management, and prevention of bronchiolitis (7). RSV infection was confirmed by direct immunofluorescence virus detection. Patients who had underlying chronic diseases (i.e., bronchopulmonary dysplasia, cystic fibrosis, interstitial lung diseases, congenital heart diseases, immunocompromised state), or co-infected with other pathogens were excluded.

### RSV Detection

Nasal or oropharyngeal specimens were collected with un-flocculated, polyester-tipped plastic swabs either in the emergency room or within 24 h after admission. Then, RSV was detected using direct immunofluorescence virus detection kits (Chemicon Intemation Inc, Temecula, CA, USA) according to the manufacturer's instructions. If the smear contains more than 200 cells, and there are more than 2% of the cells show yellow-green fluorescence in the cytoplasm, we could judge as RSV infection.

### Study Design

According to the critical parameters in the latest guideline about bronchiolitis in China (8, 9), including feeding, respiratory rate, respiratory effort, SPO_2_ as well as mental state, patients were classified as mild, moderate or severe. The clinical characteristics, laboratory data and radiological features during hospitalization were collected. Details of ongoing treatments, history of atopic diseases and previous hospitalizations were also recorded. Furthermore, demographic data were obtained from parents through structured questionnaire, including the status of pregnancy, delivery, birth, breastfeeding and weaning, exposure to tobacco smoking or pets contact, family history of allergies, and so on.

Follow-up was conducted by the same doctor at 1, 3, 5, and 7 years after discharge to monitor any episode of wheezing or even diagnosis of asthma. If there was an episode of wheezing, the details about the cause, the frequency and the process were recorded. Recurrent wheezing was defined as ≥3 episodes of wheezing but not diagnosed as asthma by a pediatrician. Asthma was defined according to the GINA recommended (10).

### Statistical Analysis

The statistical analyses were performed using IBM SPSS Statistics 20 software. Data were described by frequencies for categorical data, and by mean ± standard-deviation (SD) or median for continuous data, according to the distribution from normality. All tests were two-sided and a *P*-value < 0.05 was considered statistically significant difference. A one-way non-parametric analysis of variance (ANOVA) followed by an unpaired Student's *t*-test was conducted for comparing the parameters among groups. Enumeration data were subjected to Chi-square test or Fisher exact test where appropriate. Multivariable logistic regression was performed adjusted for clinically relevant and statistically significant factors.

## Results

### Demographic Data of Patients With RSV Bronchiolitis Among Three Groups

425 patients were initially recruited and 283 patients completed the 7-year follow-up. Among them, 17 cases were excluded due to incomplete data, and the remaining 266 cases were analyzed as following. The main reason for drop-out was the change of telephone number or the wrong registration number. Those who were loss of follow-up did not differ from others regarding gender, onset age, initial score of severity, laboratory indexes, treatment therapy, and hospital duration.

Of the 266 patients, the mean (SD) age of onset was 4.9 (3.3) months, and the male-to-female ratio was 2.5. The mean (SD) birth weight of all patients was 3.22 (0.63) kg, and the number of patients who had a history of preterm birth, cesarean section, breast feeding, and eczema was 28, 148, and 72, respectively. According to the outcome of follow-up, patients were divided into three groups. Of them, 36 cases showed recurrent wheezing (RW group), 65 cases were finally diagnosed with asthma (AS group), and the remaining 165 cases were completely recovered (CR group). Patients who developed asthma later were older than those in the CR group (*P* < 0.05), and the birth weights were much higher as well (*P* < 0.05). When comparing with the CR group, a significantly higher proportion of cesarean sections was found in the RW group (*P* < 0.05). We also found that there was a higher likelihood of allergic condition in the AS group and more tobacco exposure in the RW group, though the difference did not reach statistical significance. Other demographic data did not differ significantly among the three groups (As shown in Table 1).

**Table 1:** Demographic data of patients with RSV bronchiolitis among three groups.

**Parameters**	**All participants (*n* = 266)**	**CR (*n* = 165)**	**RW (*n* = 36)**	**AS (*n* = 65)**
Age of onset (months)^a^	4.9 (3.3)	4.5 (2.9)	5.3 (3.9)	5.7 (3.72)^*^
Gender (male/female)	190/76	119/46	25/11	46/19
Birth weight (kg)^a^	3.22 (0.63)	3.17 (0.69)	3.23 (0.55)	3.35 (0.45)^*^
Premature, *n* (%)	28 (10.53%)	21 (12.73%)	2 (5.56%)	5 (7.69%)
Cesarean section, *n* (%)	148 (55.64%)	82 (49.7%)	26 (72.22%)^*^	40 (61.54%)
Breast feeding, *n* (%)	124 (46.62%)	78 (47.27%)	17 (47.22%)	29 (44.62%)
Eczema, *n* (%)	72 (27.07%)	39 (23.64%)	9 (25.00%)	24 (36.92%)
Familial history of atopy, *n* (%)	24 (9.02%)	14 (8.48%)	2 (5.56%)	8 (12.31%)
Tobacco smoke exposure, *n* (%)	65 (24.44%)	34 (20.61%)	13 (36.11%)	18 (27.69%)
Pet exposure, *n* (%)	18 (6.77%)	12 (7.27%)	3 (8.33%)	3 (4.62%)

*Data are expressed as number (proportion) of children in each group except as noted*.

a *Data represents the mean (SD)*.

* *P-value < 0.05*.

*CR, Completely Recovered; RW, Recurrent Wheezing; AS, Asthma*.

### Clinical Characteristics of Patients With RSV Bronchiolitis Among Three Groups

Of 266 patients, 44 cases presented fever, 104 cases presented shortness of breath, and the mean cough duration and wheezing duration was 7.1 (5.5) days and 8.5 (5.5) days, respectively. According to the clinical manifestations, 158 patients were in the mild degree, 61 were in the moderate degree and 28 were in the severe degree of disease. However, there was no significant difference in the degree of severity among the three groups. As to laboratory data, we found that there was a remarkable increasing of serum IgE in the AS groups than that in the CR group (*P* < 0.01).

The treatments of patients were also shown in Table 2. 20.3% patients were received oxygen therapy, while antibiotics, systemic glucocorticoids, and ICS were given in 71.43, 43.23, and 86.09% cases, respectively. Interestingly, all patients in the RW group were applied with ICS, which meant that the incidence of using ICS was much higher than that in the other two groups (*P*_RW&CR_ < 0.05, *P*_RW&AS_ < 0.01).

**Table 2:** Clinical characteristics of patients with RSV bronchiolitis among three groups.

**Parameters**	**All participants (*n* = 266)**	**CR (*n* = 165)**	**RW (*n* = 36)**	**AS (*n* = 65)**
**Manifestations**				
Fever, *n* (%)	44 (16.54%)	25 (15.15%)	4 (11.11%)	15 (23.08%)
Cough Duration (days)^a^	7.1 (5.5)	7.1 (5.3)	5.8 (2.7)	7.6 (6.9)
Wheezing Duration (days)^a^	8.5 (5.5)	8.3 (5.7)	8.7 (3.8)	8.9 (5.8)
Shortness of breath, *n* (%)	104 (39.1%)	63 (38.18%)	12 (33.33%)	29 (44.62%)
**The degree of Severity**				
Mild, *n* (%)	158 (59.40%)	98 (59.39%)	24 (66.67%)	36 (55.38%)
Moderate, *n* (%)	61 (22.93%)	40 (24.24%)	4 (11.11%)	17 (26.15%)
Severe, *n* (%)	28 (17.67%)	27 (16.36%)	8 (22.22%)	12 (18.46%)
**Laboratory data**				
E%^a^	1.12 (1.55)	1.21 (1.61)	0.72 (1.16)	1.12 (1.56)
IgE (IU/ml)^a^	41.42 (63.63)	30.6 (39.68)	51.83 (83.08)	58.88 (85.01)^**^
**Treatments**				
Oxygen therapy, *n* (%)	54 (20.3%)	33 (20%)	7 (19.44%)	14 (21.54%)
Antibiotic usage, *n* (%)	190 (71.43%)	127 (76.87%)	28 (77.78%)	50 (76.92%)
ICS, *n* (%)	229 (86.09%)	142 (86.06%)	36 (100%)^*^/^**^	51 (78.46%)
Systemic GC, *n* (%)	115 (43.23%)	75 (45.45%)	18 (50%)	30 (46.15%)

*Data are expressed as number (proportion) of children in each group except as noted*.

a *Data represents the mean (SD)*.

* *P-value < 0.05*,

** *P-value < 0.01*.

*CR, Completely Recovered; RW, Recurrent Wheezing; AS, Asthma; GC, glucocorticoids; ICS, inhaled corticosteroid*.

### Risk Factors for Post-bronchiolitis Recurrent Wheezing or Asthma

**Figure 1:**
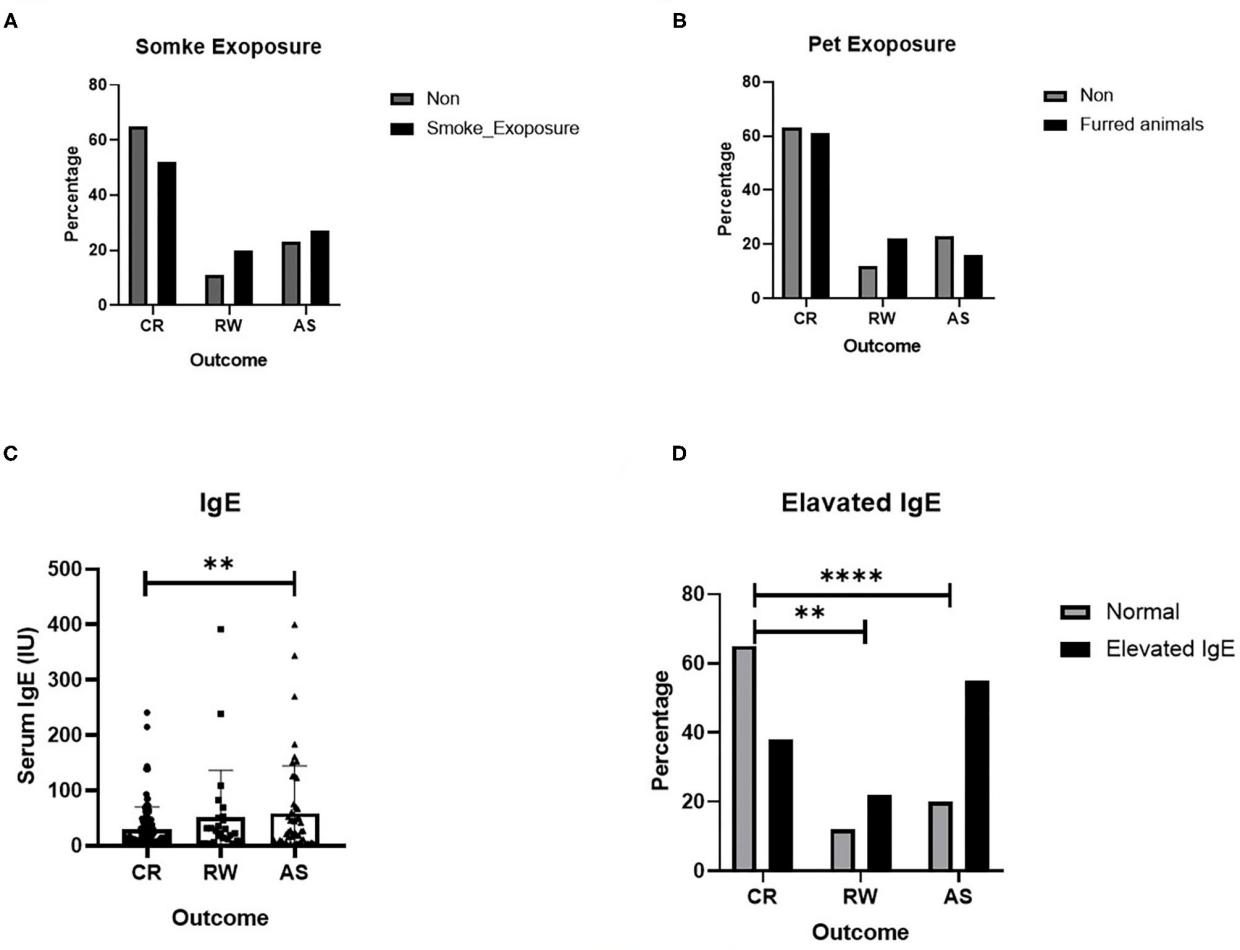
Several risk factors for recurrent wheezing and asthma. **(A)** Effect of smoke exposure; **(B)** Effect of Pet exposure; **(C)** Serum IgE level; **(D)** Effect of elevated IgE. The data are expressed as the mean ± SD or *n* (%). **P-value < 0.01, ****P-value < 0.0001.

Several possible hereditary, environmental and clinical factors for the development of recurrent wheezing and asthma were evaluated by using multiple logistic regression. Due to some blank cells, tobacco smoke and pet exposure and IgE levels were excluded in logistic regression and analyzed elsewhere, with a univariate test (Figure 1). As shown in Table 3, those delivered by cesarean section, or had an older age of onset also tend to experience wheezing repeatedly, OR equals 2.797 and 1.421, respectively. And the higher birth weight was associated with post-bronchiolitis asthma, OR equals 1.369. It is prudently to say that smoke exposure poses both risks, while pets only increase the likelihood of repeated wheezing since the intentional avoidance in children with asthma. Ultimately and pivotally, History of eczema is more directly relates to higher asthma risk than generally atopic condition, which is consistent with the serum IgE levels.

**Table 3:** Multinomial logistic regression of various risk factors for post-bronchiolitis recurrent wheezing and asthma.

**Outcome^a^**		***P*-value**	**OR**	**95% CI**	
				**Lower bound**	**Upper bound**
RW	Intercept	0.00			
	Gender	0.69	0.846	0.368	1.945
	Cesarean section	0.01^*^	2.797	1.246	6.281
	Preterm birth	0.55	1.293	0.551	3.033
	Birth weight	0.63	1.088	0.776	1.525
	First child	0.32	0.666	0.300	1.479
	Eczema	0.89	1.063	0.432	2.615
	Family allergy	0.35	0.466	0.093	2.335
	Onset age	0.05^#^	1.421	1.007	2.007
	Severity	0.85	1.052	0.626	1.767
	Fever	0.28	0.509	0.150	1.724
	Wheezing duration	0.12	0.422	0.144	1.235
AS	Intercept	0.00			
	Gender	0.40	0.750	0.383	1.469
	Cesarean section	0.15	1.559	0.849	2.862
	Preterm birth	0.72	1.154	0.527	2.528
	Birth weight	0.04^*^	1.369	1.014	1.847
	First child	0.81	0.922	0.480	1.771
	Eczema	0.09^#^	1.808	0.919	3.554
	Family allergy	0.61	1.283	0.490	3.359
	Onset age	0.10	1.265	0.960	1.667
	Severity	0.16	1.346	0.890	2.036
	Fever	0.15	1.607	0.837	3.084
	Wheezing duration	0.98	1.007	0.618	1.641

a *The reference category is: CR*.

# *P-value < 0.1*,

* *P-value < 0.05*.

*CR, Completely Recovered; RW, Recurrent Wheezing; AS, Asthma*.

## Discussion

There is no doubt that RSV is the leading cause of acute bronchiolitis, accounting for 50~80% (11). A growing number of studies have linked bronchiolitis to different outcomes. As early as 1999, Stein et al. have reported that early postnatal RSV infection was an independent risk factor for asthma, which has been supported by many clinical studies and prospective cohort studies (2, 12). However, in recent years, research on twin populations in Denmark indicates that RSV infection only increases the risk of wheezing in the short term, while long-term effects were questioned (13). Furthermore, it is well-known that asthma is affected by genes, environment and the level of economics, which would lead to regional differences in asthmatic incidence. So based on these controversies, this study focused on acute bronchiolitis caused by RSV infection with a long-term follow-up, and is committed to reinterpreting post-bronchitis recurrent wheezing or asthma from multiple perspectives. To avoid interactions or additive effects between different pathogens, patients with co-infection were excluded. In our research, the incidence of recurrent wheezing and asthma was 13.53 and 24.44%, respectively, that is, more than one-third of children who will experience repeated wheezing after bronchiolitis with RSV infection. This number is much higher than the prevalence of pediatric asthma in mainland China, which is about 3~4% (14).

Overall, most children with bronchiolitis are mild, and the severity of bronchiolitis does not appear to have a significant effect on the patient's prognosis. Herein, age is an important factor worth exploring. Most studies believe that the younger the child occurs bronchiolitis, the higher the risk of bronchial asthma (15), while other studies have concluded that there is no statistical difference in the incidence of asthma in children with bronchiolitis in different age groups (16). According to our results, an older age of onset is probably associated with a higher likelihood of asthma in the future. In the early stages of life, every month or even every day is very important for a baby's growth, but it is a pity that fewer studies are accurate to the month of RSV infection. In previous studies, the discussion about children's weight and susceptibility to asthma was contradictory. Some studies believe that lower birth weight, rather than higher birth weight, is more likely to develop asthma (17). But there are also studies claiming that excessive early life weight gain and overweight status were associated with an increased risk of asthma in childhood (18). Similarly, in our study we found asthma was more likely to occur in babies with a higher birth weight. More attention and consideration should be paid to verify the relationship between these two factors and children's prognosis.

As for the exposure of some risk factors in early life, such as cesarean section, smoke and pet exposure history will only increase the risk of repeated wheezing within a certain period of time, which is almost pre-school age, that is, about 5 years old to be exactly. Smoke exposure has long received widespread attention. It is generally believed that heavy smoking mothers in the fetal and early infant period increases the incidence of asthma, and smoking behavior in young men may even affect the respiratory health of offspring born many years later (19).

Consistent with other previous researches, post RSV bronchiolitis asthma is a closely related to allergic constitution, especially the history of eczema and serum IgE levels (20, 21). It is generally believed that the body mainly exhibits T-helper 1 (Th1) dominant immune responses when infected by a virus, while asthma manifests as T-helper 2 (Th2) dominance. Academician Zhong Nanshan had reported in the early years that BALB/c mice had Th1 type changes in the lung immune response after RSV infection alone, but when RSV infection was superimposed after OVA sensitization, they showed a bias toward Th2 responses (22). It is necessary to mention Rhinovirus (RV) here, another important cause of bronchiolitis in infants and young children, which declared to be more closely related to recurrent wheezing and even the development of asthma compared with RSV and other viral infections (23). Specifically, infantile RV infection-induced bronchiolitis and atopic constitution are risk factors for the development of allergic asthma; while RSV infection, the age of first episode of wheezing <12 months, and parents smoking are risk factors for developing non-allergic asthma (24). What is new is that the SNPs polymorphism at the 17q21 gene locus can increase the risk of early life RV infection, although some studies have suggested that this gene polymorphism is not related to atopy status (25). It reminds us to think whether a genetic susceptibility of viral infection in asthmatic children or some special viruses which do increase the risk of asthma that finally led to what we see. Microbial imbalance has also been a hot topic in recent years. It is now widely accepted that the local flora of patients with bronchiolitis is similar to asthma status (26).

In summary, RSV bronchiolitis in infants and young children undoubtedly increases the risk of recurrent wheezing or asthma compared with the general population from the same place (27). Among them, allergic constitution is an important prerequisite for the occurrence of asthma, and related risk factor such as cesarean section can only increase recurrent wheezing to a certain extent and within a certain period of time. And we also find higher birth weight and older onset age for those who develop asthma, which should be verified in the future.

## Data Availability Statement

The original contributions generated for the study are included in the article/supplementary material, further inquiries can be directed to the corresponding authors.

## Author Contributions

YZho, LT, and YZha conceptualized and designed the study. ML, YW, LL, and DY collected and analyzed the data. ZC designed the study and reviewed and revised the manuscript. All authors approved the content of the manuscript, approved the final manuscript as submitted, and agreed to be accountable for the content of the work.

## Conflict of Interest

The authors declare that the research was conducted in the absence of any commercial or financial relationships that could be construed as a potential conflict of interest.
